# Simultaneous GPS-tracking of parents reveals a similar parental investment within pairs, but no immediate co-adjustment on a trip-to-trip basis

**DOI:** 10.1186/s40462-021-00279-1

**Published:** 2021-08-21

**Authors:** Marwa M. Kavelaars, Jan M. Baert, Jolien Van Malderen, Eric W. M. Stienen, Judy Shamoun-Baranes, Luc Lens, Wendt Müller

**Affiliations:** 1 Behavioural Ecology and Ecophysiology Group (BECO), University of Antwerp, Universiteitsplein 1, 2610 Antwerp, Belgium; 2 Terrestrial Ecology Unit (TEREC), Ghent University, K.L, Ledeganckstraat 35, 9000 Ghent, Belgium; 3 Research Institute for Nature and Forest (INBO), Havenlaan 88, 1000 Brussels, Belgium; 4 Theoretical and Computational Ecology, IBED, University of Amsterdam, P.O. Box 94240, 1090 GE Amsterdam, The Netherlands

**Keywords:** Parental investment, Sexual conflict, Parental coordination, Biologging, Seabirds, Lesser black-backed gulls

## Abstract

**Background:**

Parental care benefits the offspring, but comes at a cost for each parent, which in biparental species gives rise to a conflict between partners regarding the within-pair distribution of care. Pair members could avoid exploitation by efficiently keeping track of each other’s efforts and coordinating their efforts. Parents may, therefore, space their presence at the nest, which could also allow for permanent protection of the offspring. Additionally, they may respond to their partner’s previous investment by co-adjusting their efforts on a trip-to-trip basis, resulting in overall similar parental activities within pairs.

**Methods:**

We investigated the coordination of parental care measured as nest attendance and foraging effort in the Lesser black-backed gull (*Larus fuscus*), a species with long nest bouts that performs extended foraging trips out of sight of their partner. This was achieved by GPS-tracking both pair members simultaneously during the entire chick rearing period.

**Results:**

We found that the timing of foraging trips (and hence nest attendance) was coordinated within gull pairs, as individuals left the colony only after their partner had returned. Parents did not match their partner’s investment by actively co-adjusting their foraging efforts on a trip-by-trip basis. Yet, pair members were similar in their temporal and energetic investments during chick rearing.

**Conclusion:**

Balanced investment levels over a longer time frame suggest that a coordination of effort may not require permanent co-adjustment of the levels of care on a trip-to-trip basis, but may instead rather take place at an earlier stage in the reproductive attempt, or over integrated longer time intervals. Identifying the drivers and underlying processes of coordination will be one of the next necessary steps to fully understand parental cooperation in long-lived species.

**Supplementary Information:**

The online version contains supplementary material available at 10.1186/s40462-021-00279-1.

## Introduction

By caring for their young, parents increase the growth and survival chances of their offspring and benefit from more successful reproduction, but their investment comes at a cost [1]. Parents must hence decide how to allocate the resources they gather between themselves and their offspring, while additionally coordinating their parental investment with their partner. The latter, however, is not conflict free, as both parents benefit from allocating a greater proportion of care and its associated costs to their partner [1, 2]. Concomitantly, in monogamous species with long-term pair bonds, partners should not overexploit their partner but instead cooperate [3], as they benefit if their partner remains in good condition for future breeding attempts [4].

Parental cooperation and the conflict over within-pair distribution of parental care is the subject of a longstanding and ongoing debate, forming the central framework for a broad range of behavioural studies. Initially, Houston and Davies [5] suggested that parents could simply have a fixed agreement on levels of care, a “sealed bid”, without the need to respond to each other’s investment. However, many studies show otherwise, i.e. parents modify their behaviour in response to their partner’s efforts (reviewed in [6]). Later models allowed parents to adjust levels of parental care to their partner’s investment via some form of behavioural negotiation [7–9]. The consensus of these models is that parents should invest below the most optimal level of care in order to avoid exploitation by their partner (i.e. the costs of negotiation) [7, 8, 10]. More recently, it has also been argued that parents could resolve their conflict via a reciprocal turn-taking strategy, in which parents temporally space visits to the nest in response to visits made by their partner [11]. In birds where both parents incubate eggs and provide food for young, such strategies could occur both at the incubation stage (nest attendance) and nestling stage (food provisioning), thereby minimising conflict [12] and potentially improving reproductive output [13–15]. This requires that partners monitor each other [11, 16], which may be possible in many passerine species that forage in close proximity to the nest, have short time-intervals between their nest visits, and limited variation in prey size. Yet, in most other species, the opportunities for parents to evaluate each other’s contribution is much more limited.

In many seabird species, for example, both partners are involved in parental care [17–19], which entails resource provisioning as well as protection of young. Foraging trips often last several hours to days and visits to the nest are infrequent and often irregular (e.g. [20, 21]), while leaving the nest unattended may increase the risk of predation [22]. Foraging strategies may differ between partners, as individuals of many seabird species are highly specialised on specific prey items or consistently forage in the same areas [23–28]. Given this inter-pair variability in foraging behaviour and the necessity of nest attendance, each parent thus has to adjust its foraging effort and time allocated to its offspring needs, while also considering the needs of its partner that cannot forage as long as it is protecting the nest.

Not much is known about how seabirds coordinate their levels of parental care, as initially studies had to rely on direct nest observations to study the coordination of parental activities such as nest attendance. Direct nest observations are very time intensive, especially in species with long foraging trips that result in widely spaced nest visits, but they provided first evidence for parental coordination. For example, Coulson and Johnson [29] showed that kittiwake (*Rissa tridactyla*) parents tended to remain at the nest until the foraging partner returned. Similar alternating nest visiting sequences have been observed in various other seabird species, both during incubation as well as during the chick rearing period [19, 30, 31]. Even though chicks were more likely to be left alone as they became older (see also [32]), parental nest attendance remained more coordinated than predicted by chance if partners acted independently [29]. Some of the initial technical challenges have in the meanwhile been resolved. Transponder systems that automatically detect individuals in the colony may ease the study of nest attendance, allowing researchers to measure the duration of incubation stints and time away from the nest (e.g. [29, 32]), while additionally enabling them to get a closer look at nest visiting patterns. Using transponders, Tyson et al. [33] found a pattern of foraging trips in Manx shearwaters (Puffinus puffinus) that were indicative for within-pair coordination, affirming previous suggestions for coordination in related species [34–36]. With the frequent occurrence of coordination among all these different seabird species, these studies altogether hint at the importance of cooperation between parents. However, they give little insight into how parental efforts are divided between the pair members as an important component, namely the foraging effort, is rarely studied in this context (but see [18, 37]). Observing seabirds during their foraging trips remains difficult, meaning that important information on parental investment that occurs away from the nest is missed.

Biologging provides new opportunities for studying behaviour out of sight, giving insight in both the temporal spacing of nest visits and foraging effort, even in species with extensive foraging trips. High resolution tracking data allow studying, for example, foraging strategies [38–42], diet and habitat use [43–47], making this method suitable for investigating offspring provisioning in great detail [18]. However, without following both parents at the same time, the activities of one of the parents has always been left uncertain. Simultaneously GPS-tracking both parents can provide detailed information about how coordination could be achieved in taxa where methodological challenges previously precluded detailed studies. Hence, it holds great potential for improving our understanding of parental cooperation.

**Fig. 1:**
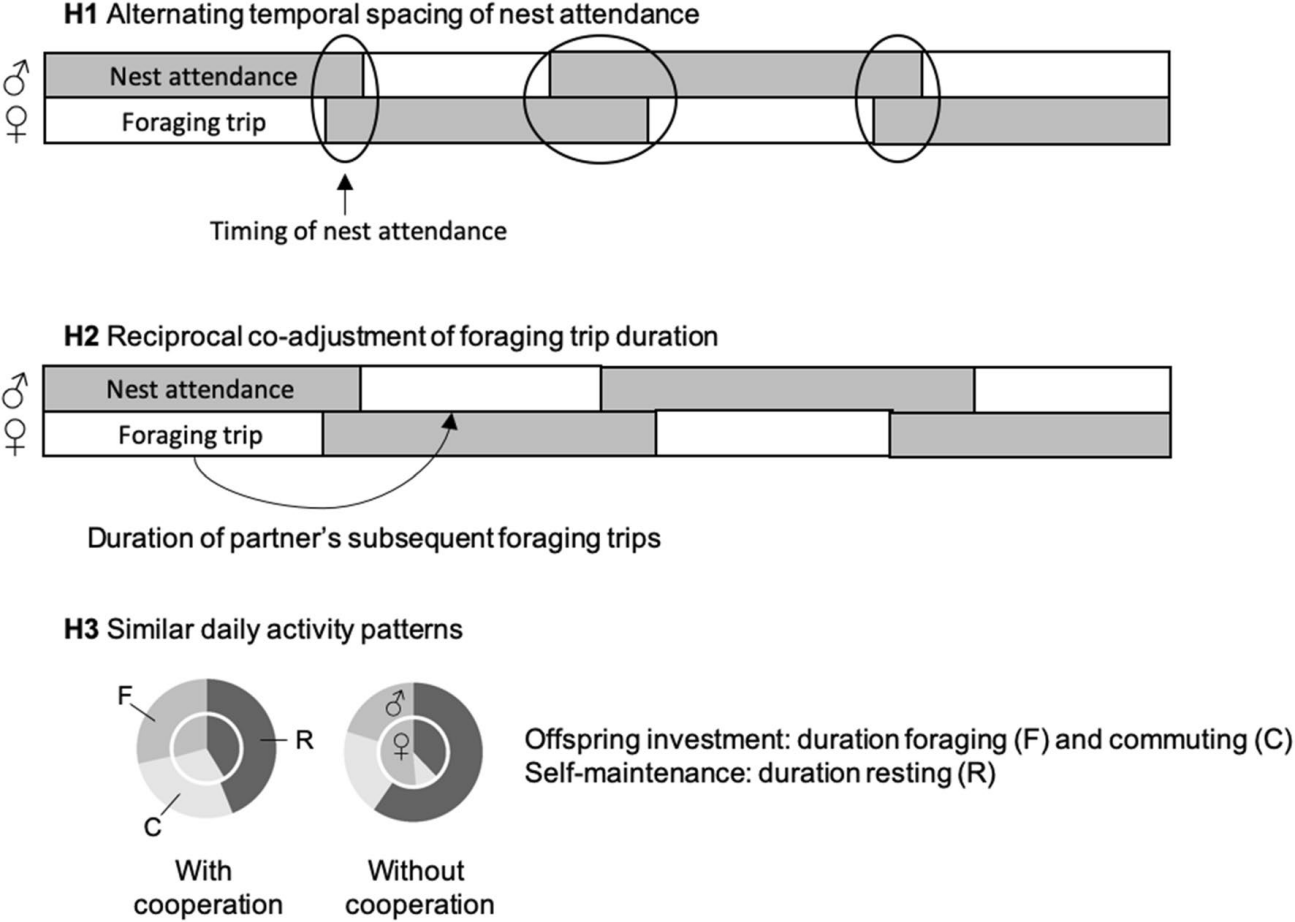
Outline hypotheses

In this study, we leveraged the advantages of biologging technology to investigate parental coordination in a generalist seabird species that performs extended foraging trips out of sight of their partner (Fig. 1). Specifically, we simultaneously GPS-tracked Lesser black-backed gull (*Larus fuscus*) parents during the entire chick rearing period, which has never been undertaken in this context before. Lesser black-backed gulls are central place foragers during the breeding season, adopting a variety of individual foraging strategies using agricultural, marine and urban habitats [28, 48, 49]. Daily activity patterns can be assumed to primarily consist of foraging, resting and commuting between foraging area and colony [50]. First, we quantified the proportion of time that at least one of the parents was present at the nest along with the likelihood that a trip started after the partner arrived in the breeding colony. We hypothesise that parents will temporally space their presence at the nest to that of their partner so that a permanent protection of the offspring is guaranteed (H1). If the coordination of foraging trips is linked to offspring guarding, we predict that parents will wait less for each other as the breeding season progresses, given that chicks become less vulnerable to predation as they grow older. Second, partners may not only coordinate their presence at the nest, which they might achieve by waiting for each other, but partners could additionally adopt a reciprocal turn-taking strategy. Besides alternating foraging trips, individuals will then also respond to their partner’s previous investment by co-adjusting foraging effort on a trip-to-trip basis (H2). Thus, we examined whether parents match the duration of their own foraging trip to the duration of the last foraging trip made by the partner. Third, such coordination of foraging behaviour could result in overall similar daily activities and possibly equal parental investment within pairs (H3). To test how parents cooperate, that is how they share their efforts, we measured how much time each parent spends foraging, commuting between breeding and foraging sites, and self-maintenance (time allocated to resting outside colony), during the entirety of the chick rearing period.

## Materials and methods

### Field work: GPS-tracking

In 2015–2017, fieldwork was carried out in the colonies of Vlissingen, the Netherlands (51° 27′ N, 3° 42′ E), and Zeebrugge, Belgium (51°20' N 3°10' E). We caught 25 pairs (respectively 13 and 12 pairs in Vlissingen and Zeebrugge) of Lesser black-backed gulls on the nest in the second or third week of incubation and deployed UvA-BiTS GPS devices on both partners using a Teflon wing harness (61 × 25 × 10 mm, 13.5 g + 5 g harness; for more detailed information on the UvA-BiTS GPS devices see [51]; for wing harness attachment see [52]). Previous work in Lesser black-backed gulls using the same GPS devices and attachment method, with one or both parents tagged, found no effect of GPS-tracking on offspring growth [53]. Neither were there any carry-over effects to the next breeding season found [54], although this does not exclude potential deleterious effects on specific behavioural traits (e.g. [55]). This biologging system enables remote collection of GPS data, allowing us to follow both parents simultaneously throughout the entire breeding season (and thereafter for different research purposes). GPS fixes were taken every three minutes. In addition, 10 s of 20-Hz accelerometer data were collected simultaneously, so that we could distinguish a number of behaviours (see below). This allowed us to derive the following parameters for our analyses: trip duration (H1, H2, H3), trip distance (H3), flight duration (H3), foraging duration (H3) and self-maintenance (resting outside colony) duration (H3).

As parents may not only respond to the behaviour of the partner, but also to offspring cues, we standardised brood size and offspring demand among the pairs, by letting the focal pairs raise two unrelated chicks. We did this by replacing the complete clutch (modal clutch size is three eggs) of focal gulls by two unrelated pipping eggs 48 h before the moment of hatching. Nests were visited every 2–3 days and monitored until fledging (day 30). Chick mortality was recorded during each visit to define the period of chick rearing and nests were followed until both chicks died or fledged.

### Data processing

For the analyses, we only used GPS data collected during the chick-rearing period, i.e. from the moment of egg hatching until the chicks fledged or the breeding attempt failed. Trips may start or end during the night, therefore we also included trips during the dark hours. For each individual, the GPS data were spliced into separate foraging trips that started with the last GPS fix inside the colony boundaries and ended with the first GPS fix inside the colony boundaries. For each foraging trip, the following parameters were calculated from hatching (day 0) until fledging (day 30) or failure: total distance covered, which is the cumulative point to point distance (total distance in km) and time away from the colony (duration in hours). See Additional file 1: *Supplementary Information* for number of days of tracking for each pair. Trips shorter than 30 min and trips less than 1 km in distance were excluded because resources close to the colony are scarce and it is unlikely that these were foraging trips, based on their short duration and distance.

### Behavioural classification

For our research aims, we considered three behaviours of interest: resting, commuting and foraging. A priori we defined resting as all truly inactive behaviour on land or at sea, excluding all apparent inactive behaviour that could be part of a sit-and-wait strategy. The latter could often be readily identified from the presence of active foraging behaviour at the same location. Similarly, commuting flights were defined as those flights in between foraging or resting sites, excluding the straight flights at sea that are typical of birds tracking fishing vessels, which were considered to be part of the foraging strategy. Foraging, thus, comprised all behaviours where birds were either actively foraging or in search of prey items (e.g. tracking a boat or walking on a field).

In order to automatically annotate behaviour to our tracking data, we used a random forest classifier. To this end, annotators were first asked to assign GPS data points on a map to the three possible behaviours (resting, commuting or foraging) based on their expert knowledge in the field and their experience with analysing tracking data. These annotations were highly consistent among the researchers involved. Subsequently, we trained and validated a variety of model structures based on the expert annotated dataset of 128 tracking days for 64 individuals. We randomly selected half of the birds (i.e. 64 days of tracking data on 32 birds) for model training, using the other half exclusively for validation. The model structures comprised of 4 different combinations of input information streams: path geometry (step length and turning angle), path geometry and habitat (Corine land cover categories, [56]), path geometry and body movement (ground speed and classification of the accelerometer profile based on a previously developed classification algorithm, [57]), or all three information streams. In addition, we considered 3 different input window sizes (1, 3, or 5 points) to accommodate information contained in the movement sequences (i.e. a focal and the one or two previous and consecutive points). For these 12 candidate input structures, we first optimised both the number of trees and tree-depth based on half of the individuals, and then validated model predictions based on the other half of the individuals. The best performing model used a combination of path geometry, habitat and body movement, as well as a moving input window of 3 points. We then calculated the model’s accuracy, Cohen’s kappa and the sensitivity (i.e. the fraction of correct positive predictions) and specificity (i.e. the number of correctly predicted instances) for each of the three behaviours of interest. The optimal model yielded an average overall accuracy of 83.2% (Cohen’s kappa 0.712). Precision/recall for commuting, foraging and resting behaviours were 96/98%, 74/90% and 79/91%, respectively. A full description of our behavioural annotation routine can be found in the Additional file 1﻿.

### Statistical analyses

All statistical analyses were performed in R [58]. The R packages *lme4* [59] and *glmmTMB* [60] were used to fit mixed effects models. We report full models following Forstmeier and Schielzeth [61]. Normality and homoscedasticity of model residuals were graphically inspected. Significance was assessed at the 95% confidence level.

#### Temporal coordination of nest visits

We first tested whether the proportion of time that at least one of the parents was present at the nest, i.e. nest attendance, was higher than expected by chance. As a null model for uncoordinated care we randomised trips and nest bouts [11, 16, 62]. To obtain a distribution of the expected values, this randomisation process was repeated 999 times. Subsequently, the true observed nest attendance was calculated with the GPS trip data, which was compared to the distribution of the expected values created by the randomisation process. We tested whether the observed values were higher than expected (one-tailed test) based on the uncoordinated randomised foraging trips, and we assumed coordination when the observed value was significantly higher than the 95th percentile of the distribution of the expected values. Secondly, we tested whether the proportion of trips that started with the partner present in the colony was higher than expected. We used the same randomisation process to look at the proportion of trips that started with the partner present at the nest. This was done for males and females separately, as well as combining the data of both partners and not taking sex into account.

Furthermore, we tested if coordination of nest visits (nest attendance, the proportion of time that the nest was attended by one of the parents) was affected by offspring age. Nest attendance data were proportional, and therefore a beta regression with a logit link function was used. Couple ID and colony were included as random effects. We additionally tested whether the time duration that individuals spent in the colony in between foraging trips, i.e. nest bouts, decreased with offspring age by fitting mixed effects models using a gamma distribution with logarithmic link. Besides offspring age (continuous variable), we also included the sex of the parent and the interaction between sex and offspring age as independent variables, and added couple ID, bird ID and colony as random effects.

#### Trip-to-trip co-adjustment of foraging activities

We investigated whether parents used information regarding their partner’s time investment to determine their own levels of parental care, by testing whether they adjusted the duration of their foraging trips to the duration of the last foraging trip made by the partner. A mixed effects model was fitted, with duration of the foraging trip as the response variable predicted by the duration of the last foraging trip of the partner, as well as sex and their interaction. Additionally, bird ID and colony were included as random effects. We used a gamma distribution with logarithmic link, because the data was strictly positive.

#### Overall co-adjustment: within-pair similarity in daily activities

To investigate similarity in parental investment between partners, we calculated several proxies for energetic investment (total distance covered during a foraging trip and time spent in active flight); time investment (total trip duration and time spent foraging); and self-maintenance (time allocated to resting outside colony). For all parameters, we calculated the within-pair and between-pair dissimilarities using Multiple Response Permutation Procedure (MRPP) with the vegan package [63], which was done separately for both study colonies to account for between-colony differences in foraging behaviour. Firstly, the dissimilarity distances between observations of pair members were calculated for all investment parameters. Subsequently, the observations were randomly shuffled amongst the pairs and dissimilarity distances were calculated for random pairs. For each parameter, this permutation procedure was repeated 999 times to get a distribution of average distances for random pairs, which enabled us to get a significance value by assessing the probability of randomly getting a smaller dissimilarity distance than the average distance for the true pairs.

## Results

### Temporal coordination of nest visits

**Fig. 2:**
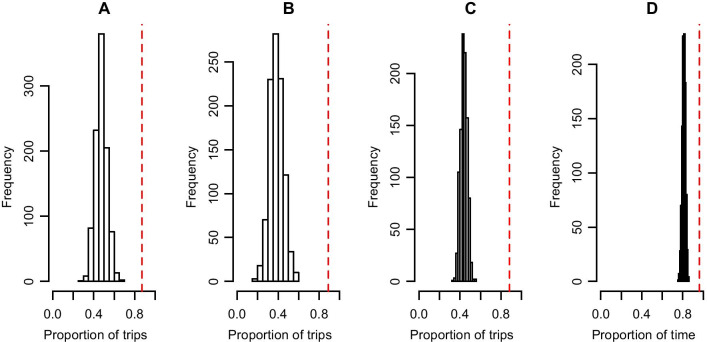
Plots comparing observed and expected values of partner presence at the nest before focal individual leaves the nest to forage using one pair as an example. Gaussian distribution of the expected values of the four different parameters (**a** female present when male leaves; **b** male present when female leaves; **c** a partner present when focal individual leaves; **d** at least one of the parents at the nest) of this pair as calculated with a randomisation process. Red dashed lines indicate observed values for that pair, which is in this case significantly higher than expected based on uncoordinated foraging trips

The proportion of trips that started after the return of the partner to the nest was higher than expected (Table 1, see Fig. 2 for an example of one pair). In males, the observed proportion of trips that started when the partner was present in the colony was 0.815, which was on average 34.2% higher than the expected values and was significant for 22 out of 25 pairs. The observed proportion of trips that females started when the partner was present in the colony was 0.834, which was on average 42.3% higher than the expected values and was statistically significant in 23 out of 25 pairs. The proportion of time that at least one of the parents was at the nest (i.e. nest attendance) was on average 0.934 and was 13.5% higher than expected, and significant for all pairs.

**Table 1:** Observed and expected values of partner presence at the nest before a focal individual leaves the nest to forage. Observed and expected values were compared for each pair

Pair	Observed proportion				Expected proportion				Difference observed and expected proportion				P value			
	Male	Female	Partner	Attendance	male	Female	Partner	Attendance	Male	Female	Partner	Attendance	Male	Female	Partner	Attendance
1	0.655	0.673	0.664	0.843	0.531	0.477	0.504	0.740	0.125	0.196	0.160	0.103	0.01	< 0.001	< 0.001	< 0.001
2	0.873	0.889	0.881	0.962	0.476	0.385	0.438	0.811	0.397	0.503	0.442	0.151	< 0.001	< 0.001	< 0.001	< 0.001
3	0.840	0.667	0.750	0.879	0.313	0.652	0.502	0.777	0.527	0.015	0.248	0.101	< 0.001	0.47	< 0.001	< 0.001
4	0.882	0.875	0.878	0.948	0.577	0.447	0.500	0.715	0.306	0.428	0.378	0.233	< 0.001	< 0.001	< 0.001	< 0.001
5	0.736	0.808	0.779	0.914	0.617	0.388	0.497	0.753	0.119	0.419	0.282	0.161	0.01	< 0.001	< 0.001	< 0.001
6	0.955	0.967	0.963	0.988	0.519	0.259	0.357	0.862	0.436	0.708	0.606	0.126	< 0.001	< 0.001	< 0.001	< 0.001
7	0.926	0.905	0.914	0.972	0.429	0.457	0.445	0.800	0.497	0.448	0.469	0.172	< 0.001	< 0.001	< 0.001	< 0.001
8	0.526	0.667	0.595	0.818	0.582	0.469	0.528	0.717	-0.055	0.198	0.066	0.101	0.86	< 0.001	0.03	< 0.001
9	0.864	0.949	0.904	0.954	0.426	0.361	0.394	0.841	0.437	0.587	0.509	0.114	< 0.001	< 0.001	< 0.001	< 0.001
10	0.842	1.000	0.912	0.989	0.512	0.295	0.387	0.832	0.330	0.705	0.524	0.157	< 0.001	< 0.001	< 0.001	< 0.001
11	0.970	0.929	0.951	0.996	0.335	0.309	0.322	0.893	0.635	0.620	0.628	0.103	< 0.001	< 0.001	< 0.001	< 0.001
12	0.800	0.789	0.793	0.983	0.37	0.296	0.323	0.889	0.43	0.493	0.470	0.094	< 0.001	< 0.001	< 0.001	< 0.001
13	0.413	0.607	0.473	0.69	0.694	0.578	0.66	0.573	-0.282	0.029	-0.187	0.117	1	0.36	1	< 0.001
14	0.861	0.809	0.832	0.985	0.31	0.342	0.327	0.892	0.55	0.467	0.505	0.093	< 0.001	< 0.001	< 0.001	< 0.001
15	0.805	0.903	0.846	0.977	0.466	0.387	0.431	0.816	0.339	0.516	0.415	0.162	< 0.001	< 0.001	< 0.001	< 0.001
16	0.943	0.952	0.948	0.979	0.409	0.415	0.412	0.822	0.534	0.537	0.536	0.157	< 0.001	< 0.001	< 0.001	< 0.001
17	0.905	0.942	0.922	0.969	0.404	0.487	0.443	0.797	0.501	0.455	0.479	0.171	< 0.001	< 0.001	< 0.001	< 0.001
18	0.847	0.772	0.801	0.937	0.407	0.359	0.378	0.852	0.441	0.412	0.423	0.084	< 0.001	< 0.001	< 0.001	< 0.001
19	0.846	0.722	0.774	0.907	0.478	0.528	0.508	0.739	0.368	0.194	0.266	0.168	< 0.001	< 0.001	< 0.001	< 0.001
20	0.535	0.635	0.582	0.833	0.561	0.482	0.526	0.722	-0.026	0.153	0.056	0.111	0.7	< 0.001	0.02	< 0.001
21	0.974	0.906	0.934	0.999	0.319	0.359	0.342	0.883	0.654	0.547	0.592	0.116	< 0.001	< 0.001	< 0.001	< 0.001
22	0.827	0.980	0.903	0.966	0.523	0.408	0.474	0.777	0.304	0.572	0.429	0.189	< 0.001	< 0.001	< 0.001	< 0.001
23	0.800	0.673	0.752	0.872	0.443	0.548	0.485	0.748	0.357	0.125	0.267	0.124	< 0.001	0.02	< 0.001	< 0.001
24	0.944	1.000	0.973	0.987	0.559	0.340	0.443	0.804	0.386	0.660	0.529	0.183	< 0.001	< 0.001	< 0.001	< 0.001
25	0.812	0.853	0.84	0.914	0.587	0.264	0.374	0.832	0.226	0.589	0.466	0.082	0.03	< 0.001	< 0.001	< 0.001
Average	0.815	0.835	0.823	0.9304	0.474	0.412	0.440	0.795	0.341	0.423	0.382	0.135				

P-value of < 0.05 means that the observed value is larger than 95^th^ percentile of distribution of expected values. Differences in observed and expected values are given for each parameter: the proportion of trips started with the female present at the nest while the male leaves (*male*), the proportion of trips started with the male present at the nest while the female leaves (*female*), the proportion of trips started with the partner present when the focal individual leaves (not taking in account sex)(*partner*), and the proportion of time that the nest is attended (at least one of the parents present the nest) (*attendance*)

**Fig. 3:**
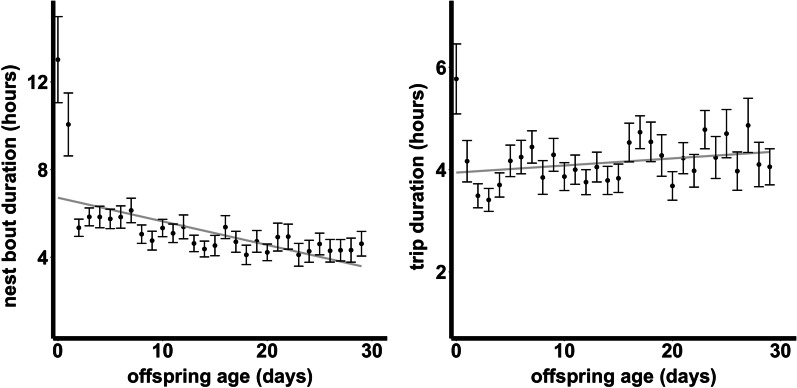
Mean ± SE nest bout duration (time spent continuously at the nest in hours) and trip duration (time spent away from the nest in hours) from hatching (day 0) to fledging (day 30). Grey trend lines are provided across all values

Nest bout duration decreased with offspring age for both males and females (− 0.0142 ± 0.005, t = − 2.617, p = 0.009, Table 2, Fig. 3). Similarly, nest attendance (proportion of time that at least one of the parents is present at the nest) decreased with offspring age (− 0.040 ± 0.005, z = − 8.687, p < 0.001, Table 2). See Additional file 1: Fig S3.1 in *Supplementary Information* for data distribution.

**Table 2:** Outcome of mixed effects models of trip duration, nest bout duration and nest attendance (proportion of time that at least one of the parents is present the nest)

		F	d.f	p-value
*Trip duration*				
Preceding trip duration partner * sex		0.1568	1	0.68
Preceding trip duration partner		0.9132	1	0.385
Sex		0.4539	1	0.521
*Nest bout duration*				
Offspring age * sex		0.2001	1	0.781
Offspring age		43.217	1	0.009
Sex		14.5944	1	0.159

		χ^2^	d.f	p-value
*Nest attendance*				
Offspring age		75.47	1	< 0.001

For trip duration and nest bout duration, a gamma distribution with log transformation was used, while for nest attendance, a beta regression with logit link function was used. The table presents the outcome of the full models. All models included bird ID and colony ID as random effects

### Trip-to-trip co-adjustment of foraging activities

The mean distance of foraging trips was 43.33 ± 0.089 km with a mean duration of 4.39 ± 0.08 h. There was no evidence that individuals adjusted foraging behaviour in terms of foraging time in response to the duration of the preceding trip by their partner, as reflected in the non-significant effect of the preceding trip duration of the partner on the trip duration of the focal individual (Table 2). This effect was independent of sex as reflected in the non-significant interaction effect. There was also no effect of sex on trip duration (Table 2).

### Overall co-adjustment: within-pair similarity in daily activities

**Fig. 4:**
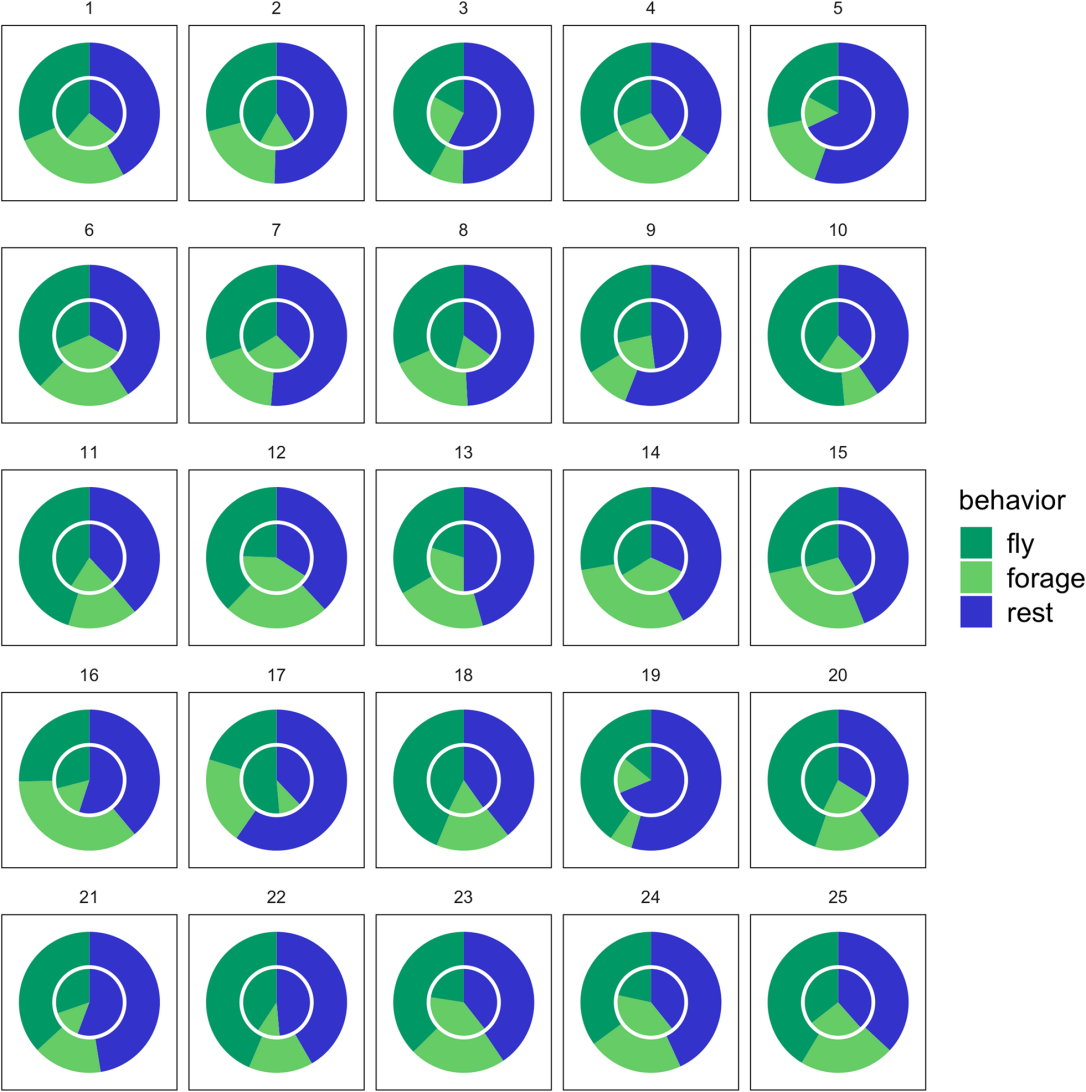
The percentage of time that individuals showed behaviour related to self-maintenance (resting outside colony) and offspring provisioning (flying and foraging), plotted for males and females of each pair separately. Female behaviour is plotted in the inner circle, male behaviour in the outer circle. Numbers refer to couple ID

Partners were more similar to each other than to other individuals of the population with regard to the estimates of the energetic and time investment, and in how much time they allocated to self-maintenance (Table 3, significance of delta < 0.001 for all parameters). These levels of investment are visualised in Fig. 4, showing that pair members, in general, invest similarly in offspring provisioning (duration flying and foraging) and self-maintenance (time allocated to resting outside colony).

**Table 3:** Outcome of a Multiple Response Permutation Procedure (MRPP) that was used to calculate the dissimilarities in offspring investment (trip duration, total distance travelled, flying and foraging) and self-maintenance (time allocated to resting outside the colony) within and between pairs

Trip parameters	Within pairs	Between pairs	Observed delta	Expected delta
*VL*				
Total distance (km)	0.223	0.252	7.133	7.895
Trip duration (h)	0.296	0.368	91.980	108.800
Resting (h)	0.231	0.266	3.117	3.481
Flying (h)	0.300	0.356	2.998	3.435
Foraging (h)	0.454	0.528	2.699	3.273
*ZB*				
Total distance (km)	0.281	0.301	8.468	9.123
Trip duration (h)	0.349	0.404	96.820	112.000
Resting (h)	0.274	0.292	3.479	3.719
Flying (h)	0.352	0.403	3.264	3.754
Foraging (h)	0.490	0.538	3.395	3.849

Dissimilarities are calculated separately for the two colonies [Vlissingen: VL (N = 12 pairs), Zeebrugge: ZB (N = 13 pairs)]. Observed delta is the overall weighted mean of group mean distances. Expected delta is the mean of original dissimilarities, under the null hypothesis of no group structure. Significance of delta is < 0.001 for all parameters

## Discussion

Simultaneously tracking the movements and behaviours of Lesser black-backed gull partners provided us with the opportunity to remotely investigate parental coordination during nestling provisioning in a species that forages out of the partner’s sight, for extended periods of time, and over long distances. Birds tended to stay at the nest until their partner returned to the colony, resulting in an alternating, temporally coordinated pattern of foraging trips. We hypothesised that joint presence at the nest may allow individuals to gain information on their partner’s previous effort to match their own foraging activity. However, we did not find evidence for a trip-to-trip co-adjustment. Intriguingly, when considering the entire chick rearing period, pair members were all in all more similar to each other than to other colony members in their daily activities including foraging, indicating that parental care is nevertheless aligned within pairs.

### Out of sight of the partner, but well-coordinated

Much of the foraging behaviour during the chick rearing phase is performed out of sight from the partner, as gulls—similar to most seabird species—travel long distances to reach their foraging grounds [64–67]. Consequently, parents can only get information on their partner’s parental investment at the nest [68] and should therefore meet at the nest between foraging trips. We found that parents normally encountered each other at the nest in between foraging trips, which is unlikely to happen if their behaviour was uncoordinated, given their lengthy foraging trips. Thus, partners responded to each other’s behaviour. Still, the question remains whether partners coordinate their contribution to care, or whether waiting for each other also serves other functions, e.g. to protect offspring from predation [69]. If the coordination of foraging trips is related to offspring guarding, it could be expected that parents wait less for each other as the breeding season progresses, since the chicks become less vulnerable to predation with increasing age [70–72]. At the same time, the offspring requirements become more demanding [73, 74] and parents may face difficulties in providing the required amount of food, especially when having to wait for the return of the partner [75]. We, indeed, found that the time parents spent at the nest in between foraging trips decreased with increasing offspring age (Fig. 3, see also [31, 34]). Note how the nest bout duration is particularly long in the first two days after hatching, as the chicks presumably need continuous brooding at this stage. The increasing proportion of time that the nest is unattended with offspring age furthermore suggests that the protection against predation could be a key driver of parental coordination in this species.

### Trip-to-trip negotiation about the levels of investment?

Above we argued that parents may have to change their nest attendance strategy over time as they have to provide less protection and more food to their offspring. Yet, at the same time, trust in the partner’s willingness to contribute to care may have grown [10], so that it becomes less relevant that parents encounter each other to co-adjust foraging activities. Previously, coordination of offspring provisioning, e.g. by synchronising [76] or alternating foraging trips [11, 16, 62] has been shown to promote equality in parental care as well as the resolution of sexual conflict [11, 12]. Thus, our findings could still be in line with the hypothesis that by alternating their foraging trips, parents could monitor each other’s efforts and potentially co-adjust their own provisioning behaviour accordingly [7, 10, 11, 16, 62, 77].

However, contrary to our expectations for a reciprocal turn-taking strategy and unlike many other species [11, 16, 62], Lesser black-backed gull parents did not match their own foraging activity to that of their partner on a trip-to-trip basis (Table 2). So, while they have information about their partner’s trip duration, they may use other cues to balance their efforts, e.g. visually inspect food transfer to the offspring. But not all food brought back to the nest is regurgitated directly after return (pers. observation), and the gulls might thus not be able to accurately estimate their partner’s effort before leaving the nest. Additionally, they may not know how much effort went into collecting food for the offspring. Parents could still use indirect information to estimate the partner’s investment via offspring begging and adjust their parental efforts accordingly (e.g. [77–81]). However, this requires the parent to know its own previous investment, how much time elapsed during which the partner should have fed, and estimate how much the partner has fed. And to further complicate matters, offspring may exaggerate their need in order to trigger parents to provide more care [82]. Moreover, if food availability is fluctuating, a single foraging trip may be less informative, and if the number of foraging trips per day is limited, any misconceived co-adjustment would have a very strong impact. Thus, in gulls and many other seabirds, a trip-by-trip co-adjustment is unlikely to be adaptive. This might be less problematic in passerines with high visit rates and limited variation in prey size, species that took the central stage when studying turn-taking strategies [11, 16]. Consequently, our current understanding of parental coordination might be biased, at least to some extent.

### Balanced efforts all in all: within-pair similarity

Furthermore, we assessed whether partners co-adjusted reproductive investment during the chick rearing period by comparing their daily activities, such as foraging, commuting and self-maintenance (time allocated to resting outside colony). We hypothesised that if partners coordinated their care and co-adjusted their reproductive investment, this should result in similar daily activities. We found that pair members were indeed more similar to each other than to other individuals of the population in both time investment (duration of trips, time spent foraging) and effort (distance travelled, time spent flying) (Table 3, Fig. 4). Parents did not seem to burden their partner with a greater proportion of the costs of parental care. Instead, pair members spent an equal amount of time on offspring provisioning (foraging and flying) and self-maintenance (resting). This is in line with a number of studies in seabirds and other taxa [83–87] that suggest that both sexes tend to invest equally during the early phase of reproduction. However, one needs to bear in mind that the costs of foraging might differ between pair members because of slight size differences related to sexual dimorphism in this species or due to other quality differences [88]. It would, therefore, be interesting to investigate how individual differences in space use influence the similarity in parental investment. In our study colony, we previously demonstrated that gull pairs that evenly distribute their care during incubation were more successful in raising offspring than pairs with a greater disparity in early reproductive investment [89]. This suggests, in combination with the findings of our current study, that a parental strategy is favoured in which pair members coordinate their efforts.

How pair members adjust their investment during offspring provisioning remains to be elucidated, as we did not find any indication of a reciprocal turn-taking strategy (see above). Negotiation about the levels of care may have already taken place at an earlier point during the reproductive attempt [5]. Previous studies have found evidence for consistency and equality in the distribution of parental care during incubation [85, 89] and demonstrated that the division of care during the early reproductive phase is maintained throughout the breeding attempt [90]. The negotiation about the care levels might thus start well before the beginning of reproduction or even go back to previous breeding events [4, 91].

For the interpretation of our results, it also important to consider that pair members respond to a shared environment, i.e. chicks having the same demand and hunger levels [92, 93]. Thereby, they may unintentionally distribute their parental efforts equivalently [69]. Furthermore, similarity in daily activities within pairs may be the result of assortative mating, as through mate choice, individuals may end up with similar-quality partners [94, 95].

## Conclusions

We found that the staging of foraging trips during offspring provisioning was well coordinated within pairs, and foraging efforts were matched within pairs, even though we did not find evidence for a direct, active co-adjustment on a trip-by-trip basis. Not instantly co-adjusting their foraging trip to their partner’s effort seems to indicate that parental cooperation does not require constant negotiation. This could be adaptive if information on the partner’s effort is unreliable or difficult to assess within short time frames, or if resource availability and thus foraging success is variable. It also implies that if parents negotiate about their respective contribution to parental care, this must take place at an earlier stage in the reproductive attempt and pair members continue to invest at the same level thereafter. Identifying the drivers and underlying processes of coordination and the equality in care levels will be one of the next necessary steps to fully understand parental cooperation in long-lived species.

## Supplementary Information

**Additional file 1.** Supplementary Information.

## Data Availability

The dataset supporting the conclusions of this article is available on 10.5281/zenodo.5213704.
