# Supplementary material for: Simultaneous GPS-tracking of parents reveals a similar parental investment within pairs, but no immediate co-adjustment on a trip-to-trip basis
Source: Mov Ecol. 2021 Aug 21;9:42. doi: 10.1186/s40462-021-00279-1 (PMC8379723; doi:10.1186/s40462-021-00279-1)
Supplement: Supplementary file 1 — Additional file 1. Supplementary Information. [file 40462_2021_279_MOESM1_ESM.docx]

**S1 Sample size tracking data**

**Table S1.1** Days of tracking data for each pair. Pairs were followed until their young fledged (days of tracking = 30) or failure of the brood (days of tracking < 30).

| Pair | days of tracking |
| --- | --- |
| 1 | 29 |
| 2 | 18 |
| 3 | 21 |
| 4 | 19 |
| 5 | 29 |
| 6 | 25 |
| 7 | 30 |
| 8 | 30 |
| 9 | 30 |
| 10 | 30 |
| 11 | 30 |
| 12 | 23 |
| 13 | 30 |
| 14 | 30 |
| 15 | 30 |
| 16 | 28 |
| 17 | 17 |
| 18 | 17 |
| 19 | 23 |
| 20 | 30 |
| 21 | 23 |
| 22 | 30 |
| 23 | 22 |
| 24 | 30 |
| 25 | 28 |

**S2 Automatic behaviour annotation**

**Lesser black-backed gull foraging ecology**

Lesser black-backed gulls use different sites for resting and foraging, hence daily activity patterns can be assumed to primarily consist of foraging, resting (including incubating and brooding during the breeding season), and commuting between foraging and resting sites (C. Camphuysen, 2013). Gulls from our three study colonies adopt a variety of foraging strategies (Figure S2.1) (Baert et al., 2018). In agricultural areas, they generally forage on foot, either by trampling for earthworms in meadows or by picking up invertebrates and small vertebrates that become exposed by ploughing or mowing in fields. Foraging is typically intermitted with short flights to move to a new spot (Camphuysen 2013). When foraging at sea, they typically track shipping vessels scavenging in flight on fishery discards intermitted with short periods of floating at sea (C. J. Camphuysen, 1995; Sotillo, Depestele, Courtens, Vincx, & Stienen, 2014). Finally, in urban and industrial settings, they often apply a sit-and-wait strategy to exploit anthropogenic food sources of which the availability is less predictable (Huig, Buijs, & Kleyheeg, 2016).

**Tracking data**

For each of the adult 64 Lesser black-backed gulls tracked during the 2015-2018 breeding seasons, we randomly selected two days of data for expert annotation. The number of tracked individuals varied among colonies, but were fairly equally distributed among sexes with 14 males and 12 females tracked from the Vlissingen colony (﻿51°27′N, 3°42′E), 14 males and 19 females from the Zeebrugge colony (﻿51°20′N, 3°10′E) and 3 males and 2 females tracked from the Ostend colony (51°13'N, 2°55’E). All individuals were equipped with 5CDLe UvA-BiTS trackers, collecting both GPS and accelerometer data (Bouten et al. 2013). Tracker settings varied between years and colonies, but were either set at a 3- or 5-minute resolution outside of the colony, and at a 20-minute resolution inside the colony. For each GPS position, 1-10s of accelerometer data was collected at 20Hz.

**Data annotation**

Data annotation was done using a custom-built R shiny app (Chang, Cheng, Allair, Xie, & McPherson, 2018), where tracks were shown on an interactive Esri leaflet (Chang et al. 2018) to provide the annotators with information on path geometry, land use and body movements (Figure S2.2). For the latter, annotators were given both the first second of the accelerometer profile as well as its interpretation based on a previously developed random forest classifier (Shamoun-Baranes, Bouten, Van Loon, Meijer, & Camphuysen, 2016), classifying the accelerometer profile as either ‘flapping flight’, ‘soaring flight’, ’extreme flapping flight’, ’mixed flight’, ’walking’, ’standing’, ’pecking’, floating’, ’sitting on a boat’, or ‘other behaviours’.


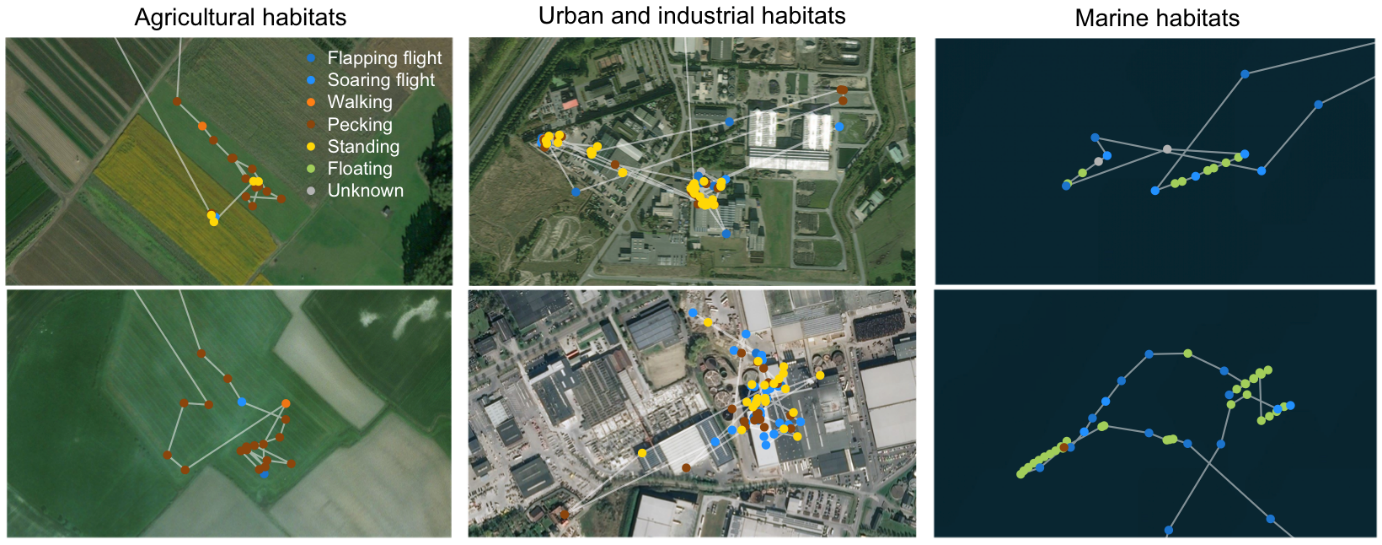


**Figure S2.1** GPS tracks illustrating the main foraging strategies used by Lesser black-backed gulls. In agricultural areas, gulls tend to hunt on foot for (in)vertebrates. In urban and industrial areas, they adopt a sit-and-wait strategy when foraging on garbage. At sea, gulls follow fishing vessels to feed on discards. GPS positions are coloured based on the behavioural classification of the accelerometer profile.

Annotators were asked to assign data points to 3 possible behaviours: resting, commuting or foraging, which comprises the main daily activities. Data points could thereby be left unannotated if the interpretation was ambiguous. We a priori defined ‘resting’ as all truly inactive behaviour on land or at sea, excluding all apparent inactive behaviour that could be part of a sit-and-wait strategy. The latter could often be readily identified from the presence of active foraging behaviour at the same location (Figure S2.1). Similarly, commuting flights were defined as those flights in between foraging or resting sites, excluding the straight flights at sea that are typical of birds tracking fishing vessels, which were considered to be part of the foraging strategy. Foraging, thus, comprised all behaviours where birds were either actively foraging or in search of prey items (e.g. tracking a boat or waiting on the roof near a container on an industrial site; Figure S2.1). Annotations were highly consistent among the 5 researchers involved (Figure S2.3).

**Random forest classifiers**

We considered 3 sources of information for behaviour classification: path geometry, habitat type, and body movement (Table S2.2). We characterised path geometry by the step length between consecutive points and the turning angle between consecutive steps. Habitat type associated with each GPS position was determined using the CORINE 2016 land cover dataset (European Environment Agency, 2016). CORINE provides a three-level classification system (level three being the most detailed). However, here, we only used the first classification level, which comprises 5 categories, as this already enabled discriminating between all habitats of interest (i.e. urban, agriculture and marine habitats, Table S2.2). Body movement was characterised based on the instantaneous ground speed of the animal derived from the acceleration data and the classification of the first second of the 3-D acceleration profile based on a previously developed random forest classifier (Shamoun-Baranes et al. 2016) (10 classes: ‘flapping flight’, ‘soaring flight’, ’extreme flapping flight’, ’mixed flight’, ’walking’, ’standing’, ’pecking’, floating’, ’sitting on a boat’, or ‘other behaviours’). Finally, as body movements such as flying or standing were not unique to each of the behaviours of interest, movement sequences could provide an important additional form of information. We therefore considered input windows of 1, 3 and 5 points (i.e. a focal point and one or two previous and consecutive points), resulting in a total of 12 candidate models (4 different combinations of path geometry, land cover and body moment information and 3 different input window sizes, Figures S2.4 and S2.5).

| **Information stream** | **Metrics** | **Values** |
| --- | --- | --- |
| **Path geometry** | Step length | numeric value in km |
|  | Turning angle | numeric value in radians |
| **Land cover** | Corine 2016 land cover value | 1 (urban surfaces),  2 (agricultural areas),  3 (forests and semi natural areas),  4 (wetlands) or  5 (water bodies) |
| **Body movement** | Instantaneous ground speed | numeric value in m s^-1^ |
|  | Classification of the accelerometer profile | ‘flapping flight’, ‘soaring flight’, ’extreme flapping flight’, ’mixed flight’, ’walking’, ’standing’, ’pecking’, floating’, ’sitting on a boat’, or ‘other behaviours’ |

**Table S2.2** Overview of random forest input parameters


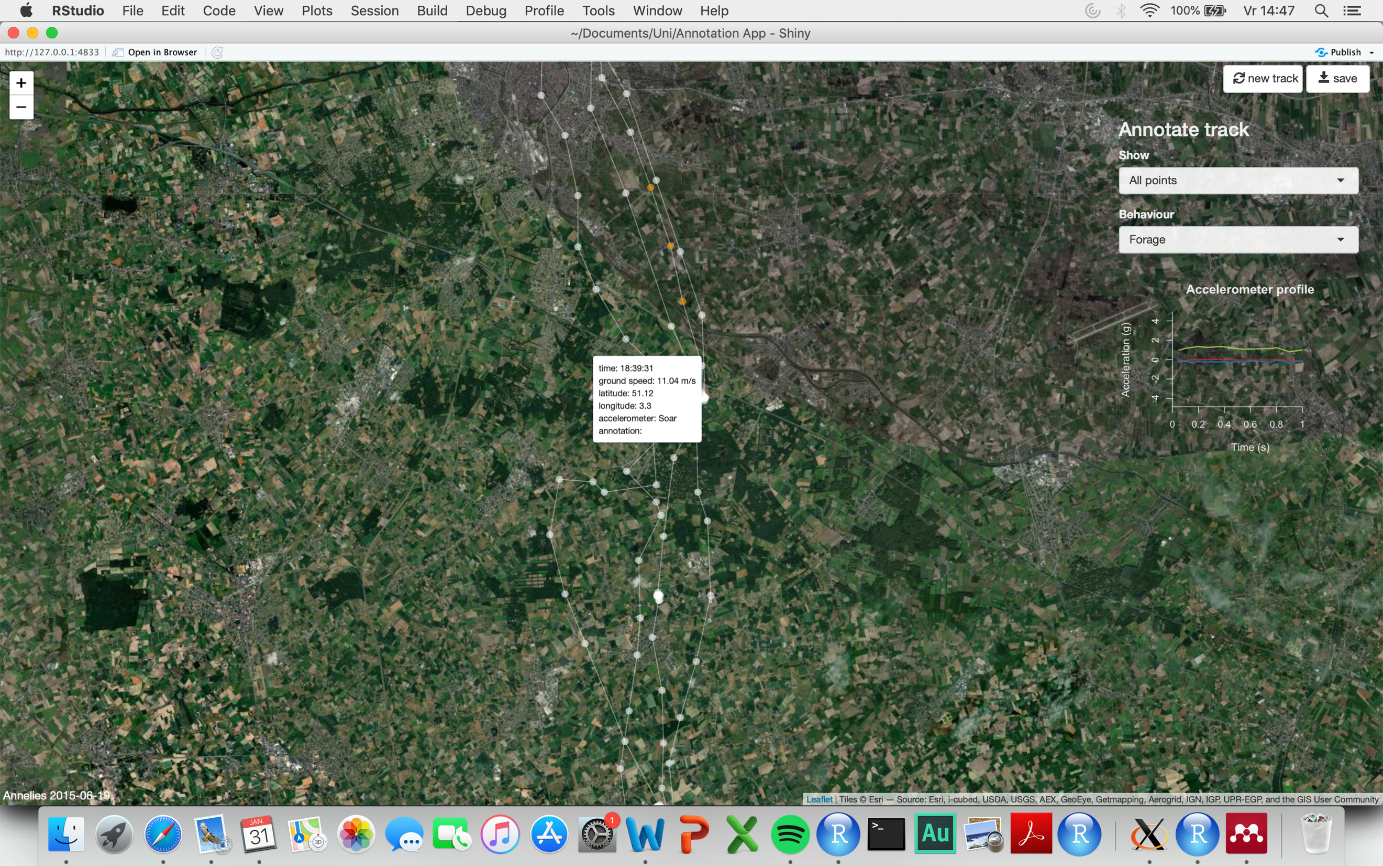


**Figure S2.2** Screenshot of the R shiny app used for data annotation. Tracks are represented on Esri leaflet tiles. When a point is selected by the annotator, a line plot of a 1s accelerometer reading is depicted (right), and a pop-up window opens giving the interpretation of the accelerometers signal based on a previously developed classifier (Shamoun-Baranes et al. 2016), the time of the day and the instantaneous ground speed.


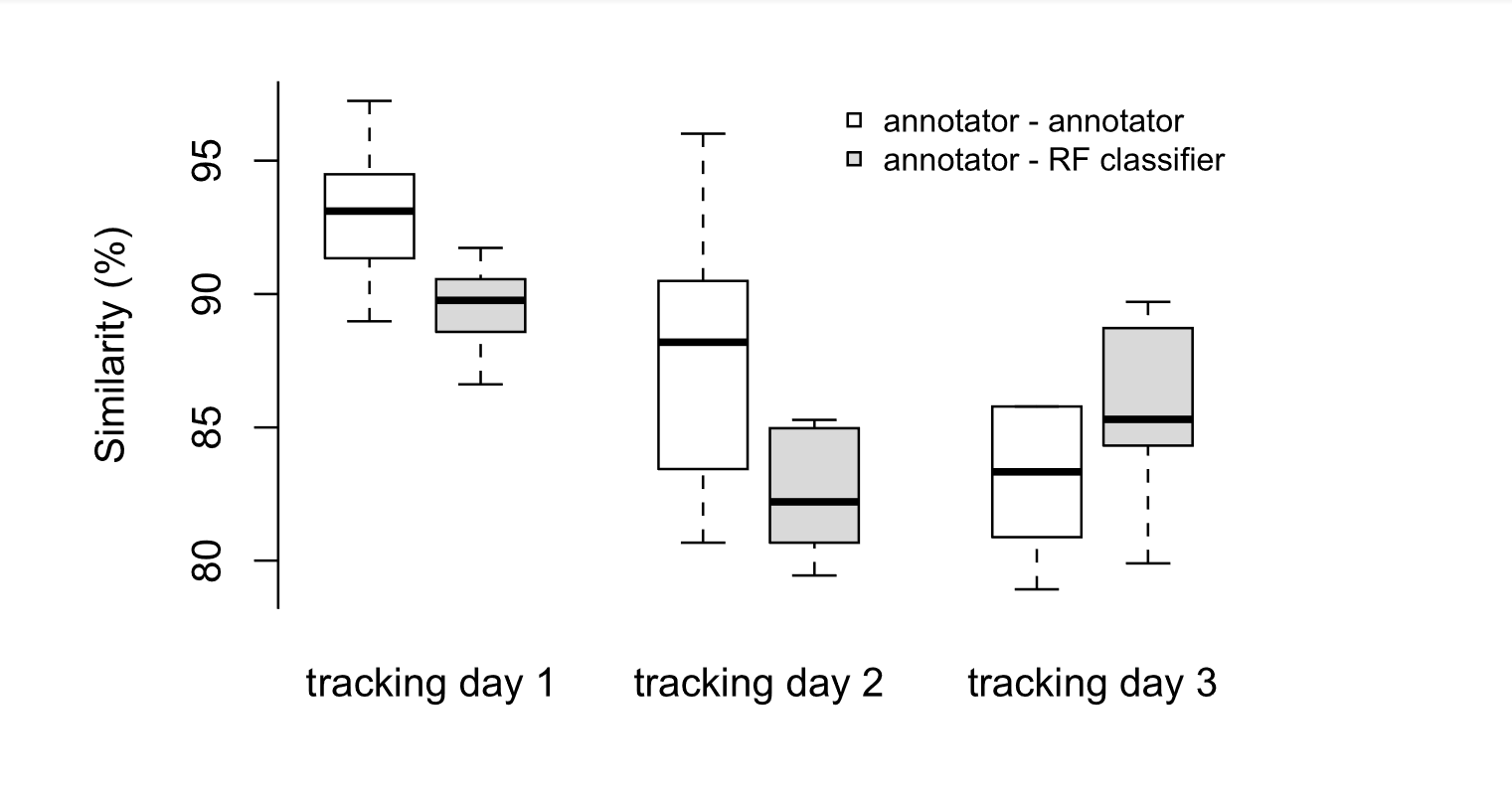


**Figure S2.3** Similarity in data annotation between 5 annotators (white boxes) and between annotators and the prediction of the RF classifier using a three-point moving input window and all data streams (grey boxes) for 3 randomly selected days of tracking data. Boxplots represent the 1st, 2nd and 3rd quartile. Whiskers correspond to maximal 1.5 times the interquartile range.

**Model regulation, training and validation**

We randomly selected half of the birds (i.e. 64 days of tracking data on 32 birds) for model training, using the other half exclusively for validation. For each of the 12 candidate input structures, we first optimized the number of trees and tree depth. We trained random forests containing 10 to 100 trees with tree depth ranging from 2 to 30 edges and selected the optimal model structure based on the out of the bag score. Next, for each of the 12 input structures, we validated model predictions of the optimal model based on the other half of the birds. For each of these 64 days of tracking, we calculated the model’s accuracy, Cohen’s kappa (Figure S2.4) and the sensitivity (i.e. the fraction of correct positive predictions) and specificity (i.e. the number of correctly predicted instances) for each of the three behaviours of interest (Figure S2.5).

**Evaluating model performances and selecting a final model**

All random forest classifiers were able to reliably identify and discriminate behaviours, with overall mean performances ranging between 73 and 82% accuracy (Figure S2.4), which is very similar to variations among researchers in manual data annotation (Figure S2.3). Both multi-point input windows and additional data streams strongly increased model performance (Figures S2.4 and S2.5). This was driven by a better ability to identify foraging and resting behaviour. Indeed, while commuting flights are readily identifiable from their particularly large step lengths and low turning angles, reliably identifying resting and foraging behaviour benefited from additional information on land cover and body movement. This is not unsurprising given the large variety in foraging strategies (Figure S2.1), and their partial overlap in path geometry with resting behaviour and commuting flights (see also ‘Lesser black-backed gull ecology’). Best performing models thus used all three information streams in a multi-point input window. However, expanding the input window from 3 to 5 points barely improved model performance. In addition, models with a 5-point input window are more prone to missing data (accelerometer data are not collected when battery power is low).

**Figure S2.4** Boxplots of model accuracy and Cohen’s kappa value for the 64 days of tracking data used for model validations. Boxplots represent the 1st, 2nd and 3rd quartile. Whiskers correspond to maximal 1.5 times the interquartile range.

**Figure S2.5** Boxplots of model sensitivity and specificity for the three behaviours of interest, for the 64 days of tracking data used for model validations. Boxplots represent the 1st, 2nd and 3rd quartile. Whiskers correspond to maximal 1.5 times the interquartile range.

**S3 Nest bout duration and trip duration**

**
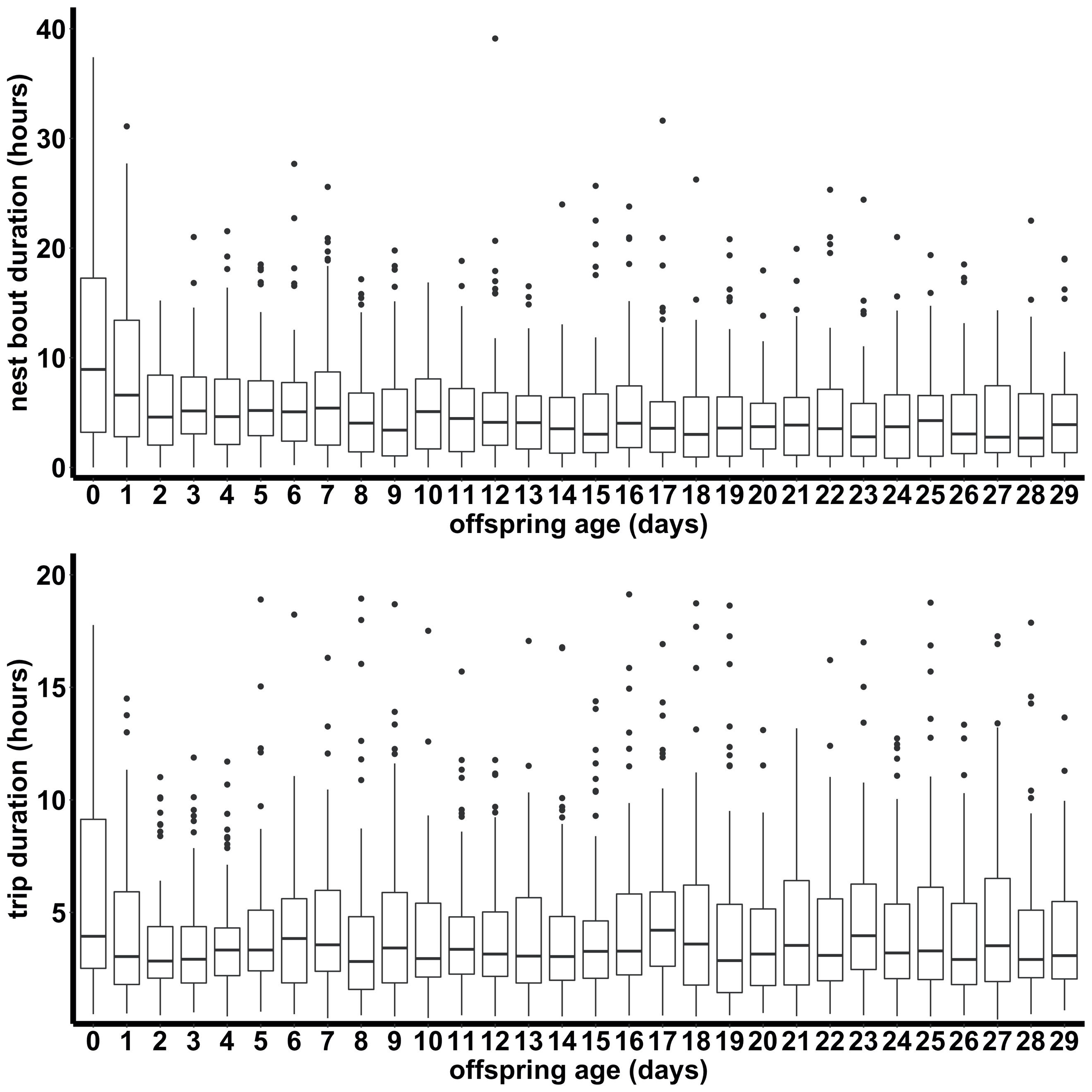
**

**Figure S3.1** Median (thick horizontal lines), 50% range (boxes), minimum and maximum value (whiskers) and outliers (points) are given for nest bout duration (hours) and trip duration (hours) in relation to offspring age (days).

**References**

Baert, J. M., Stienen, E. W. M., Heylen, B. C., Kavelaars, M. M., Buijs, R.-J., Shamoun-Baranes, J., … Müller, W. (2018). High-resolution GPS tracking reveals sex differences in migratory behaviour and stopover habitat use in the Lesser Black-backed Gull Larus fuscus. *Scientific Reports*, *8*(1), 5391. doi: 10.1038/s41598-018-23605-x

Camphuysen, C. (2013). *A historical ecology of two closely related gull species (Laridae): multiple adaptations to a man-made environment*.

Camphuysen, C. J. (1995). Herring Gull Larus argentatus and Lesser Black-backed Gull L. fuscus feeding at fishing vessels in the breeding season: competitive scavengng versuss efficient flying. *Ardea*, *83*, 365–380.

Chang, W., Cheng, J., Allair, J. J., Xie, Y., & McPherson, J. (2018). *Shiny: web apllication framework for R*.

European Environment Agency. (2016). CORINE Land Cover.

Huig, N., Buijs, R.-J., & Kleyheeg, E. (2016). Foraging herring Gulls Larus argentatus on the Dutch coast: City slickers or true seagulls? *Limosa*, *89*(2), 58–66.

Shamoun-Baranes, J., Bouten, W., Van Loon, E. E., Meijer, C., & Camphuysen, C. J. (2016). Flap or soar? How a flight generalist responds to its aerial environment. *Philosophical Transactions of the Royal Society B: Biological Sciences*, *371*(1704). doi: 10.1098/rstb.2015.0395

Sotillo, A., Depestele, J., Courtens, W., Vincx, M., & Stienen, E. W. M. (2014). Consumption of Discards by Herring Gulls *Larus argentatus* and Lesser Black-Backed Gulls *Larus fuscus* off the Belgian Coast in the Breeding Season. *Ardea*, *102*(2), 195–206. doi: 10.5253/arde.v102i2.a9
